# Exploring the mechanism of Acanthopanax in treating vertigo: A network pharmacology and molecular docking study

**DOI:** 10.1097/MD.0000000000049677

**Published:** 2026-07-10

**Authors:** Rui-Qiong Ba, Jin-Mei Ning, Ping Li, Xiang Ma, Yao Cui, Hen-Jie Zhu, Shu-Ji Gao, Yan-Lin Zhu, Ming-Wei Liu

**Affiliations:** a Department of Neurology, Qujing Central Hospital, Qujing, Yunnan, China; b Department of Otorhinolaryngology, Qujing Central Hospital, Qujing, Yunnan, China; c Department of General Medicine, Qujing Central Hospital, Qujing, Yunnan, China; d Department of Cerebrovascular Disease, Qujing Central Hospital, Qujing, Yunnan, China; e Department of Emergency, The First Affiliated Hospital of Kunming Medical University, Kunming, Yunnan, China; f Department of Emergency, Dali Bai Autonomous Prefecture People’s Hospital, Dali, Yunnan, China.

**Keywords:** Acanthopanax, molecular docking, network toxicology, target, vertigo

## Abstract

Acanthopanax has therapeutic efficacy against vertigo; however, the underlying mechanism remains unclear. This study aimed to elucidate the mechanism by which Acanthopanax treats vertigo through integrated network pharmacology and molecular docking techniques, and retrieved all target genes of Acanthopanax for vertigo treatment from July to October 2025. Vertigo-related target genes were subsequently identified from public databases, including GeneCards and Online Mendelian Inheritance in Man. The intersection between Acanthopanax-derived targets and vertigo-related targets was analyzed to identify candidate target genes. Using the STRING platform, we constructed protein-protein interaction networks for the identified candidate targets and mined the core functional modules within these networks. Gene Ontology and Kyoto Encyclopedia of Genes and Genomes enrichment analyses were performed on candidate targets via the clusterProfiler package. A carp bile poisoning-liver injury target-pathway network was constructed via Cytoscape 3.8.2 software, network topology analysis was conducted, and the core components and targets were screened. The results found that A total of 295 candidate targets for the treatment of vertigo caused by Eleutherococcus senticosus were identified. Pathway enrichment analysis revealed that Eleutherococcus senticosus treatment for vertigo may be closely associated with pathways related to IL-17, *TNF*, phosphoinositide 3-kinase (PI3K)-Akt, p53, HIF-1, and Forkhead box O signaling. The core targets for the treatment of A. senticosus vertigo include *TP53*, *AKT1*, *STAT3*, *TNF*, and JUN. Network pharmacology and molecular docking studies suggest that A. senticosus may treat vertigo by regulating targets such as JUN, *TNF*, *AKT1*, *STAT3*, and *STAT3* through pathways such as the IL-17, *TNF*, phosphoinositide 3-kinase-Akt, p53, HIF-1, and Forkhead box O signaling pathways. These mechanisms warrant further investigation in future o and in vitro studies.

## 1. Introduction

Vertigo is a common neurological disorder that is primarily characterized by disturbances in spatial orientation and balance. Mild cases may present with visual blurring or dizziness, whereas severe cases involve persistent spinning sensations and an inability to stand, significantly impacting daily life and work.^[1]^ Vertigo is a clinically prevalent and common disorder that can occur at any age and tends to recur, affecting the patients’ ability to perform normal daily activities and work. In severe cases, it may progress to syncope or stroke, potentially threatening the patient life. Research indicates that Vertigo patients often exhibit impaired vascular endothelial function and disrupted secretion of vasodilators and vasoconstrictors in the circulation. This affects cerebral vascular elasticity, leading to narrowing or spasm of cerebral blood vessels and triggering vertigo symptoms.^[2]^ NO has strong vasodilatory effects, whereas endothelin-1 (ET-1) has a potent vasoconstrictive effect. Under normal conditions, these 2 substances interact to maintain dynamic equilibrium in vascular morphology. However, in pathological states, ET-1 becomes dominant, potentially causing vasospasms. This, in turn, exacerbates cerebral hypoxia and ischemia, stimulating endothelial cells to synthesize more ET-1 and worsening vertigo symptoms.^[3]^ Current symptomatic treatments focus on improving local microcirculation, vasodilation, and neural function regulation. Betahistine is an effective medication for treating vertigo. It works by antagonizing H3 receptors to dilate capillaries, enhance microcirculation, and increase cerebral, cardiac, and peripheral blood flows.^[4]^ Simultaneously relax the presphincter muscles of the inner ear capillaries to enhance the vestibular and cochlear blood supply. It also increases capillary permeability to promote extracellular fluid absorption, alleviate endolymphatic hydrops, and control vertigo symptoms. However, symptoms often recur after discontinuation, and long-term use has multiple adverse effects, making it difficult to achieve clinically optimal outcomes.^[4]^

Acanthopanax senticosus, the rhizome or stem of the plant Acanthopanax senticosus (Rupr. et Maxim.) is a member of the Araliaceae family that tonifies qi, strengthens the spleen, nourishes the kidneys, and calms the spirit. It is primarily used clinically to treat spleen–lung qi deficiency, kidney deficiency with soreness and pain in the lower back and knees, deficiency of the heart and spleen, insomnia, and forgetfulness.^[5]^ Pharmacological studies have indicated that Acanthopanax senticosus injection dilates cardiovascular and cerebral blood vessels, reduces myocardial oxygen consumption, improves microcirculation, and scavenges oxygen free radicals, demonstrating the unique advantages of traditional Chinese medicine in preventing and treating cardiovascular and cerebrovascular diseases.^[5]^ Current clinical observations indicate that Acanthopanax senticosus has therapeutic efficacy against vertigo, although its mechanism remains unclear. In this study, we employed network pharmacology and molecular docking to explore the mechanism underlying the use of Acanthopanax senticosus for the treatment of vertigo, providing a theoretical foundation for further investigations of this mechanism through in vivo and in vitro experiments.

## 2. Data sources and research methods

### 2.1. Collection of Acanthopanax chemical constituents and target prediction

Highly credible Eleutherococcus senticosus proteins were identified as candidate genes by logging into the BATMAN-TCM database (http://bionet.ncpsb.org.cn/batman-tcm/). The compiled targets were calibrated using UniProt (https://www.uniprot.org/) data, excluding nonhuman genes and removing invalid duplicate targets to obtain standardized gene names.

### 2.2. Acquisition of vertigo-related targets

By searching the GeneCards (https://www.genecards.org/) and Online Mendelian Inheritance in Man (https://www.omim.org/) databases via the keyword “vertigo,” disease-related targets were identified. All targets from both databases were consolidated into an Excel spreadsheet, duplicates were removed, and the data were validated against the UniProt database to obtain target gene information for the disease.

### 2.3. Results of drug–disease target prediction

The obtained drug-component targets were mapped to disease targets, and a Venn diagram was created to identify the intersecting genes. Subsequently, Cytoscape 3.8.2 software was used to construct a “drug-component-action target” network. The top 5 core components ranked by degree value were caffeic acid, quercetin, S-adenosyl-l-methionine, sesamin, and ursolic acid.

### 2.4. Construction of target protein interaction networks

To further investigate protein–protein interactions in Acanthopanax senticosus treatment for vertigo, drug-intersecting genes were uploaded to the STRING interaction database (https://string-db.org/) for protein–protein interaction network construction. The species was set to “*Homo sapiens*,” with a minimum interaction score of 0.9 to ensure study credibility. All other parameters remained at default settings. The results were saved in the TSV format. The TSV file was imported into Cytoscape 3.8.2 for network analysis (Cytoscape→Tools→Network analyzer→Networkanalysis→Analyze network). The network analysis results were saved, with node size and color representing degree values; larger nodes indicate higher degree values. The edge thickness indicates the combine score magnitude, with thicker edges corresponding to higher scores. Core targets were selected to construct a protein interaction network diagram.

### 2.5. Analysis of GO enrichment and KEGG pathway data

The intersecting genes were imported into the Drug_Disease. txt. document. The RStudio org.Hs.e.g.db package was used to convert the intersecting gene symbols to Entrez IDs. Gene ontology (GO) enrichment and Kyoto ncyclopedia of enes and enomes (KEGG) pathway analyses were subsequently performed on target genes using the clusterProfiler package. Species were set to humans, with *P* < .05, as the enrichment screening threshold. The top 10 enriched terms were visualized using the ggplot2 package in RStudio. The functional roles of target proteins in drug-mediated vertigo treatment across 3 domains were annotated: biological process (BP), cellular component (CC), and molecular function (MF). KEGG pathway enrichment analysis was conducted to elucidate the signaling pathways associated with these therapeutic targets.

### 2.6. Molecular docking

In general, the higher the degree value in a network, the more important its position is. That is, proteins with higher degree values in protein interaction networks play a crucial role in vertigo drug therapy. Molecular docking between the active ingredients and key targets was performed using AutoDock Vina (1.1.2) to validate their interaction activities. The specific methods are as follows: Compounds in mol2 format are downloaded from the TCMSP official website, imported into Chembio3D for energy minimization, and then imported into AutodockTools-1.5.6 for hydrogenation, charge calculation, charge assignment, and rotatable bond configuration before being saved in “pdbqt” format; key target proteins are downloaded from the PDB database (http://www.rcsb.org/), which prioritizes human proteins and those with high structural similarity between the original ligand and the active ingredient to be docked, selecting high-resolution structures; and the protein is imported into PyMoL (2.3.0) to remove the original ligand and water molecules. The protein was then imported into AutoDockTools (v1.5.6) for hydrogenation, charge calculations, charge assignment, and atom-type specification. The data were saved in “pdbqt” format. POCASA 1.1 was used to predict protein binding sites, and the grid box size was set to 60 × 60 × 60 (0.375 Å spacing per grid point), with all other parameters at default settings. Interaction mode analysis was performed using PyMOL 2.3.0. and Kinetic simulations were conducted for the best-docking small molecules and proteins.

### 2.7. Molecular dynamics (MD) simulation

Conventional molecular dynamics (MD) simulations were performed using the GROMACS software package (version 2022.03) to investigate the binding interactions between the compound and target protein. The compound was parameterized using the Antechamber Python Parser Interface to conform to the amber14sb.ff force field parameterization. The protein complex and ligand were dissolved in an octahedral box, and system charge neutralization was achieved by adding 0.150 M sodium and chloride ions. The system was preliminarily prepared for subsequent equilibration through 50,000 energy minimization steps using the steepest descent method. Subsequently, simulations were performed in NVT (constant number of particles, volume, and temperature) and NPT (constant number of particles, pressure, and temperature) ensembles with positional constraints applied to heavy atoms. Each ensemble was subjected to 50,000 simulation steps. The system temperature and pressure were maintained at 300 K and 1 bar, respectively, to ensure adequate equilibration under the desired conditions. Following the equilibration phase, a 100 ns MD simulation was conducted without constraints, saving trajectory data every 20 ps, including energy and coordinates. The hydrogen bond patterns within the trajectory were analyzed using the hydrogen bond analysis plugin in Visual MD. Additionally, the open-source software ChimeraX (version 1.6.1) was used to visualize and analyze the interaction patterns between the compound and the protein.

## 3. Results

### 3.1. Prediction of active components and targets in Eleutherococcus senticosus

Using the BATMAN-TCM database, 17 components of Acanthopanax senticosus were identified under the conditions of a score cutoff > 0.99 (LR = 1626) and *P* < .05.

### 3.2. Vertigo-related targets

**Figure 1.**
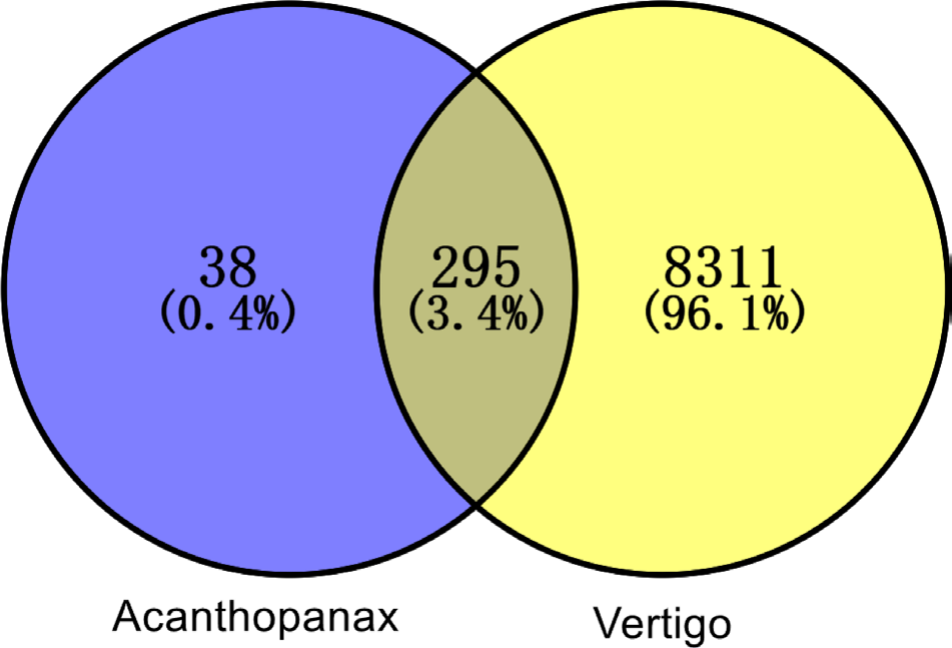
Venn diagram of interaction targets regulated by active components of Acanthopanax senticosus in vertigo.

After merging the GeneCards and Online Mendelian Inheritance in Man databases, a total of 8605 targets associated with vertigo were identified. The identified genes were validated using UniProt database. As shown in Figure 1, the intersection between drug action targets and vertigo target genes yielded 295 overlapping target genes for both vertigo and drugs, representing shared target genes for drug-based vertigo treatment.

### 3.3. Drug-component-target prediction results

**Figure 2.**
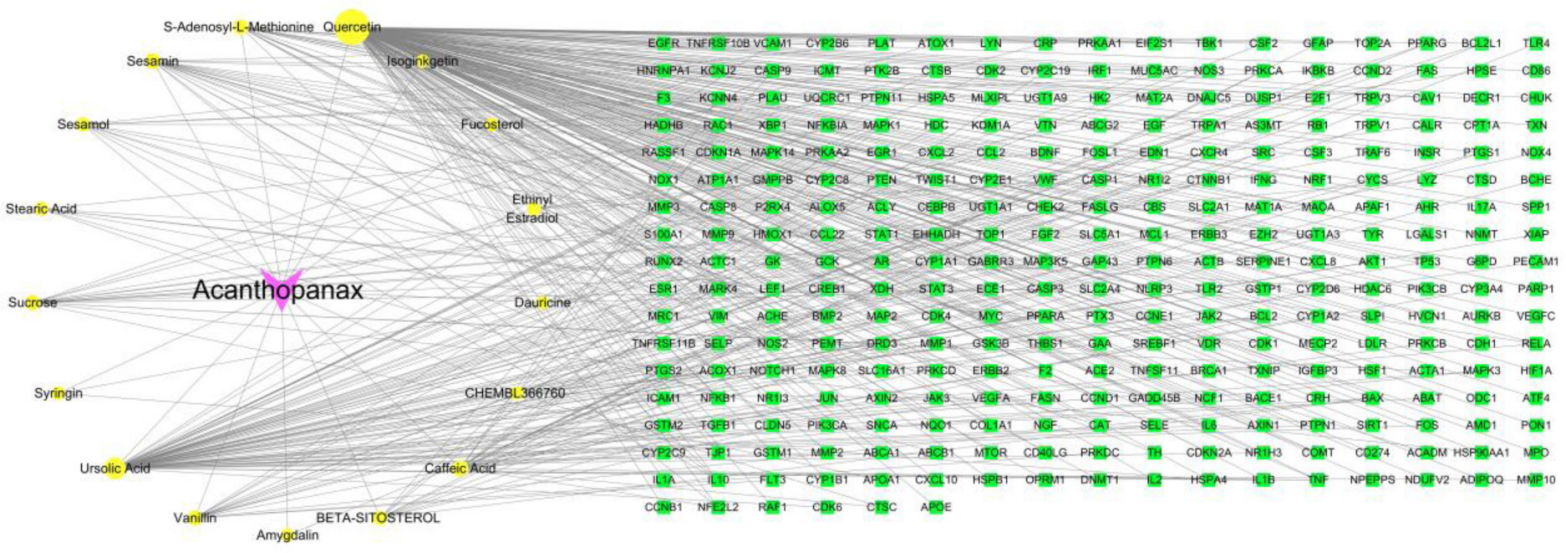
Disease-component-pathway-target network diagram. Rectangles represent targets, ellipses denote drug components, and diamond nodes indicate traditional Chinese medicine.

Using drug-component-target data, “network.xlsx” and “type.xlsx” files were constructed and imported into Cytoscape 3.7.2. As shown in Figure 2, the network comprised 313 nodes and 430 edges. The top 5 core components were caffeic acid, quercetin, S-adenosyl-L-methionine, sesamin, and ursolic acid.

### 3.4. Core targets and network interactions

**Figure 3.**
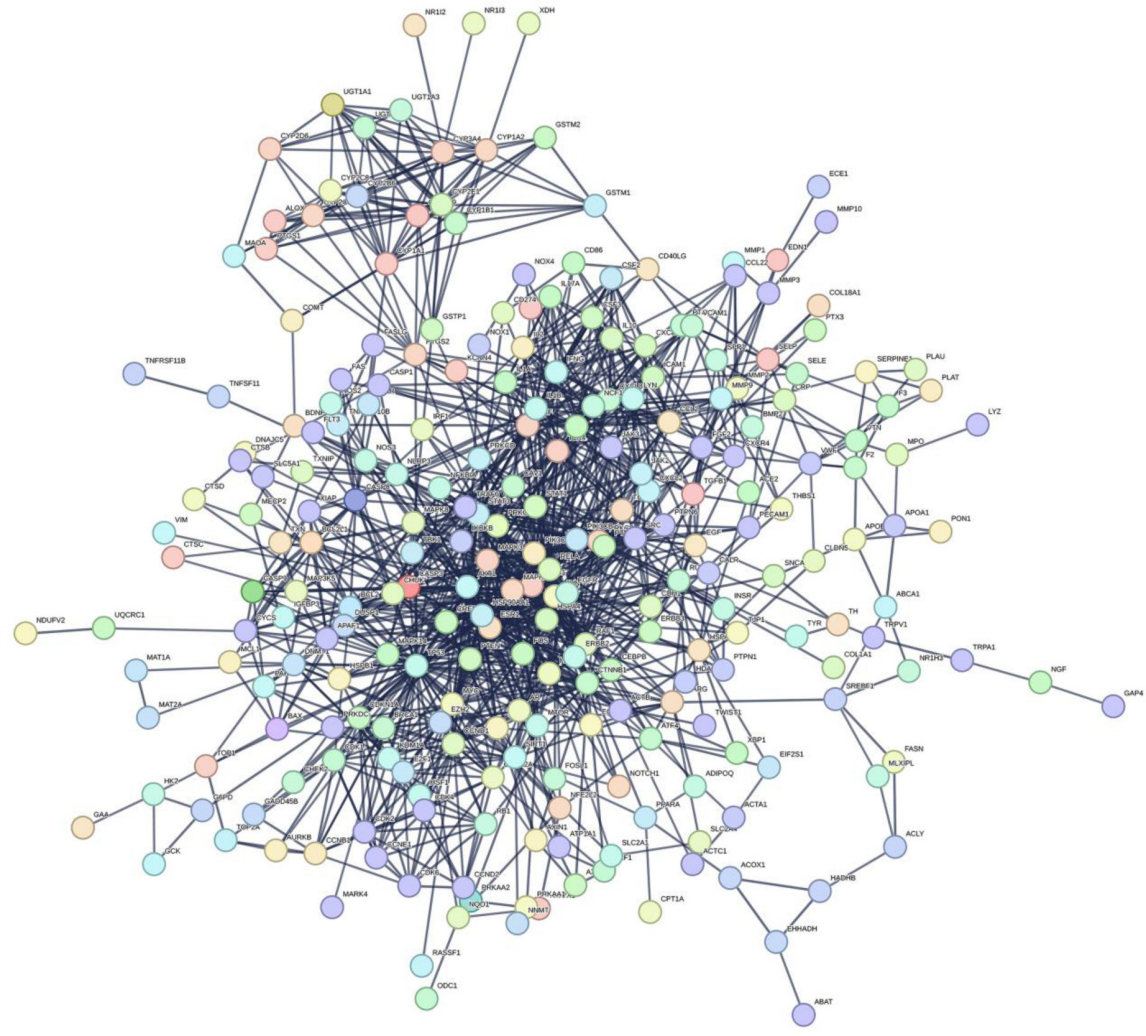
PPI network of active components in Eleutherococcus senticosus regulating vertigo.

**Figure 4.**
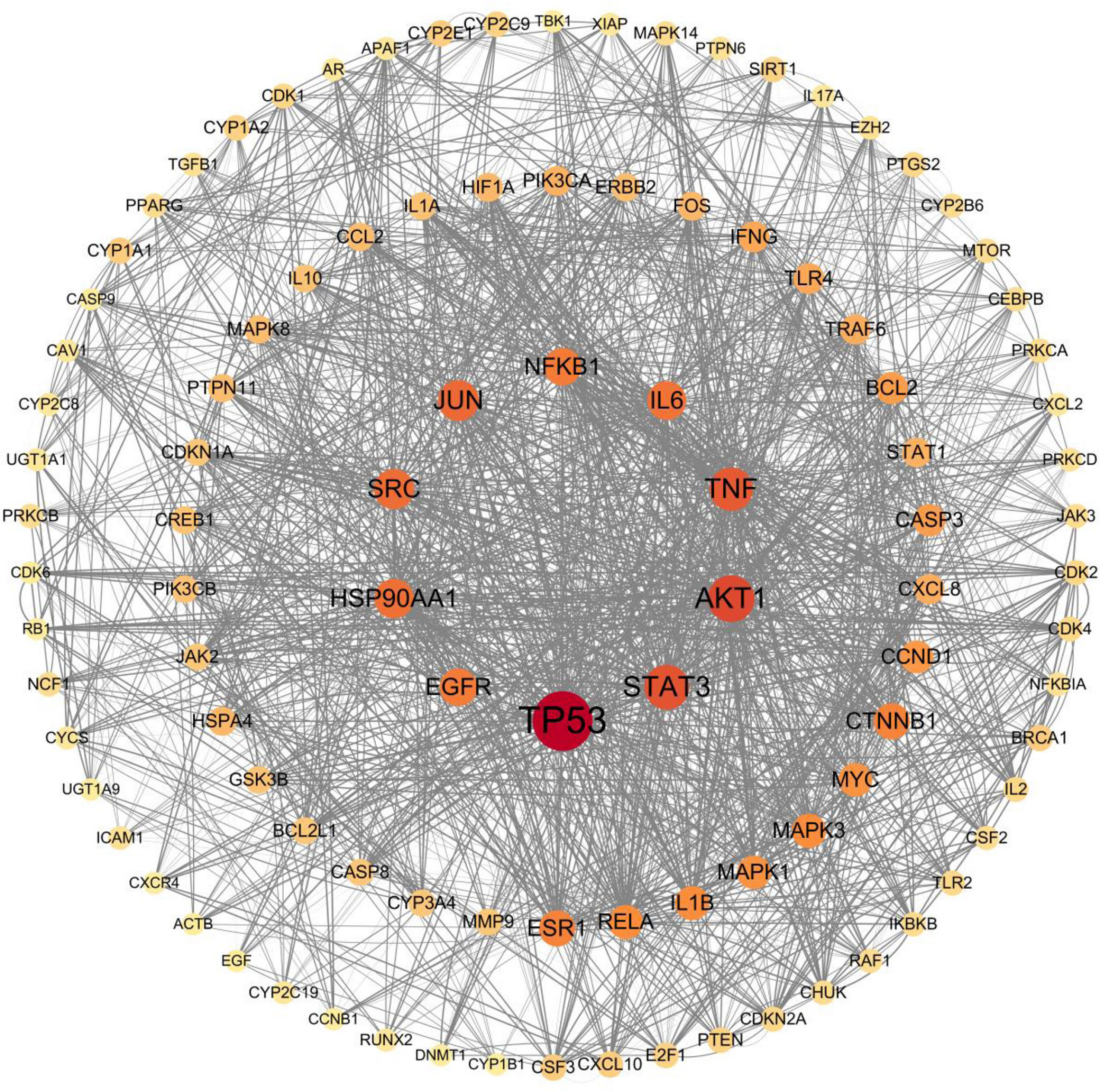
Core targets of active components of Acanthopanax in the regulation of vertigo. Rectangles represent targets, ovals denote drug components, and diamond nodes indicate traditional Chinese medicines.

After all drug action targets were intersected with vertigo target genes, 295 overlapping target genes for vertigo and drugs were identified, serving as shared target genes for drug-based vertigo treatment. The 295 intersecting target genes were imported into the STRING database (https://string-db.org/) for protein–protein interaction prediction, with the species set to *Homo sapiens* and the confidence threshold set to 0.9. The network file was saved in TSV format and imported into Cytoscape 3.7.2, to visualize the protein interaction network (Fig. 3). Target genes with a degree value >9 were selected, resulting in a network comprising 102 nodes and 1524 edges. As shown in Table 1, topological analysis was performed on the network, where the degree values indicated the target node size and color, and combined score values were used to determine edge thickness, thereby constructing the protein–protein interaction network (Fig. 4). The key targets included *TP53*, protein kinase B (*AKT1*), signal transducer and activator of transcription 3 (*STAT3*), tumor necrosis factor (*TNF*), JUN, proto-oncogene tyrosine-protein kinase SRC (SRC), HSP90AA1, IL-6, epidermal growth factor receptor (*EGFR*), and nuclear factor kappa B subunit 1 (NF-kB1)(Fig. 4).

**Table 1:** Topological property parameters of key targets in the treatment of vertigo by Acanthopanax.

Name	Degree	Average shortest path length	Betweenness centrality	Closeness centrality	Clustering coefficient	Is single node	Eccentricity
*TP53*	67	2.15354331	0.20395694	0.46435101	0.14382632	FALSE	6
*AKT1*	47	2.25984252	0.07193955	0.44250871	0.18963922	FALSE	5
*STAT3*	44	2.2480315	0.07946205	0.44483363	0.2167019	FALSE	5
*TNF*	42	2.33070866	0.06845135	0.42905405	0.24041812	FALSE	5
JUN	38	2.24015748	0.10172789	0.44639719	0.27027027	FALSE	5
SRC	37	2.39370079	0.07206645	0.41776316	0.23273273	FALSE	5
HSP90AA1	36	2.38976378	0.04535265	0.4184514	0.18412698	FALSE	6
IL6	35	2.43307087	0.03459769	0.41100324	0.3092437	FALSE	6
*EGFR*	33	2.47244094	0.03251627	0.4044586	0.22159091	FALSE	6
NFKB1	33	2.38188976	0.03457675	0.41983471	0.28030303	FALSE	6

### 3.5. Analysis of biological function enrichment

#### 3.5.1. GO enrichment analysis

**Figure 5.**
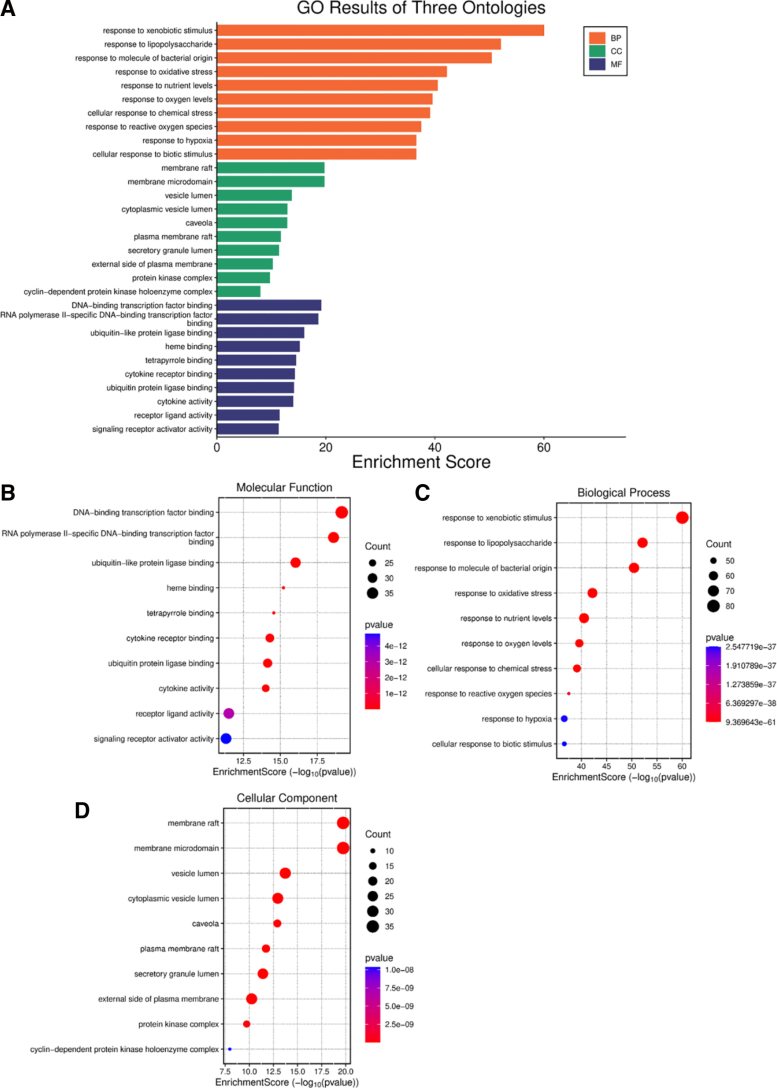
GO functional enrichment analysis diagram of the active components of Eleutherococcus senticosus that regulate vertigo-related intersecting genes. (A) GO results for 3 ontologies: (B) Biological process diagram; (C) Cellular component diagram; (D) Molecular function. GO = gene ontology.

Enrichment analysis using the clusterProfiler package identified 3823 GO terms, including 3369 related to BP, 160 related to cellular components (CC), and 294 related to MF. The top ten GO terms for BP, CC, and MF were selected for visualization. As shown in Figure 5, biological processes primarily involve responses to exogenous stimuli, lipopolysaccharides, bacteria-derived molecules, oxidative stress, nutrient levels, oxygen levels, cellular responses to chemical stress, reactive oxygen species, hypoxia, and cellular responses to biological stimuli. The enriched cellular components primarily included membrane rafts, membrane microdomains, vesicle lumen, cytoplasmic vesicle lumen, pits, plasma membrane rafts, secretory granule lumen, plasma membrane exterior, protein kinase complexes, and cyclin-dependent protein kinase holoenzyme complexes. The enriched MF included binding to DNA-binding transcription factors, RNA polymerase II-specific DNA-binding transcription factors, ubiquitin-like ligases, heme, tetrapyrrole, cytokine receptors, ubiquitin ligases, cytokine activity, receptor–ligand activity, and signal receptor activator activity.

#### 3.5.2. KEGG pathway enrichment analysis

**Figure 6.**
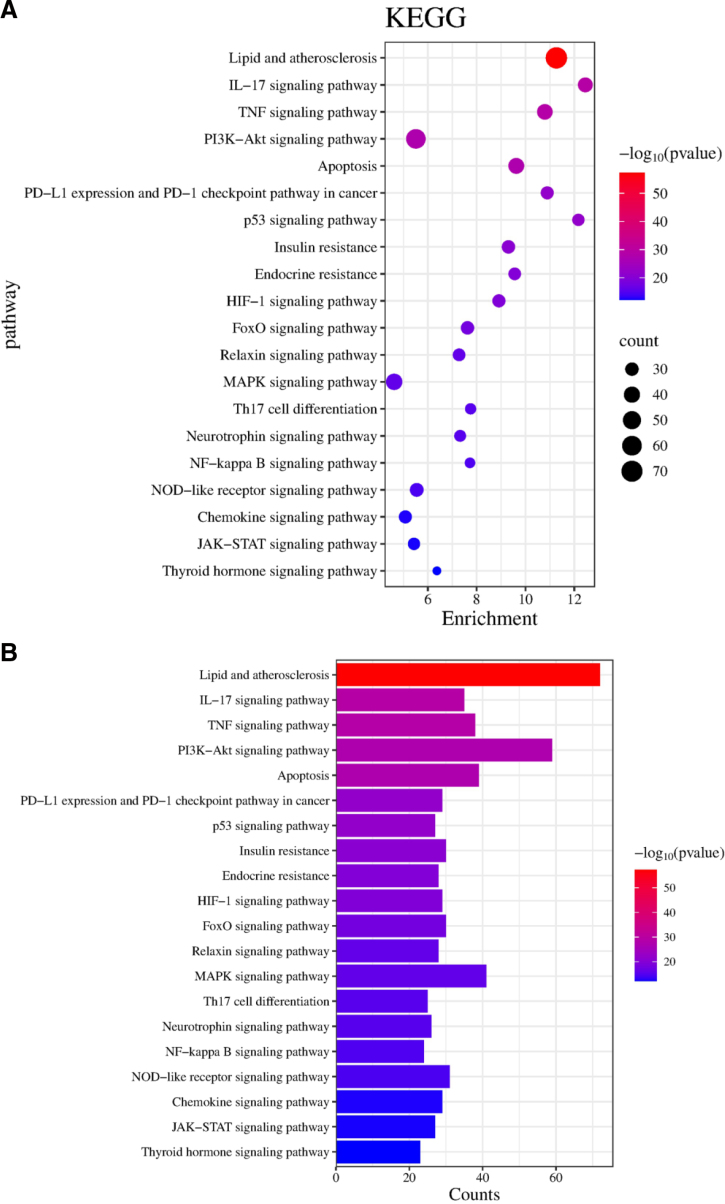
KEGG functional enrichment analysis diagram of the active components in Eleutherococcus senticosus that regulate genes associated with vertigo. Circle size represents data enrichment of genes in corresponding pathways, with shades from green to red indicating progressively decreasing *P*-values. On the basis of the *P*-values, the top 20 KEGG metabolic pathways were selected and visualized as a bubble chart. The horizontal axis represents the number of genes enriched in each pathway. Bubble size indicates gene enrichment quantity, whereas color intensity reflects statistical significance, enabling intuitive observation of significant enrichment patterns. KEGG = Kyoto Encyclopedia of Genes and Genomes.

KEGG pathway analysis identified 107 pathways involved in drug-treated lymphomas. The top 20 pathways were selected for visualization, as shown in Figure 6. Key pathways included lipid metabolism and atherosclerosis, IL-17 signaling, *TNF* signaling, phosphoinositide 3-kinase (PI3K)-Akt signaling pathway, apoptosis, programmed death-ligand 1 expression in cancer, PD-1 checkpoint pathway, p53 signaling pathway, insulin resistance, endocrine resistance, HIF-1 signaling pathway, Forkhead box O (FoxO) signaling pathway, vasopressin signaling pathway, mitogen-activated protein kinase (MEK) signaling pathway, Th17 cell differentiation, neurotrophin signaling pathway, NF-κB signaling pathway, NOD-like receptor signaling pathway, chemokine signaling pathway, JAK-STAT signaling pathway, and thyroid hormone signaling pathway. These findings suggest that drugs may regulate and intervene in diseases via these pathways.

### 3.6. Results of molecular docking

Based on the preceding analysis, the top 5 key targets and compounds with higher affinity values were selected for semi-rigid docking. The binding affinity between the small molecule and target protein is represented by the binding energy. A binding energy of <0 indicates that the small molecule can freely bind to the target protein, with lower values indicating a greater likelihood of binding.

**Figure 7.**
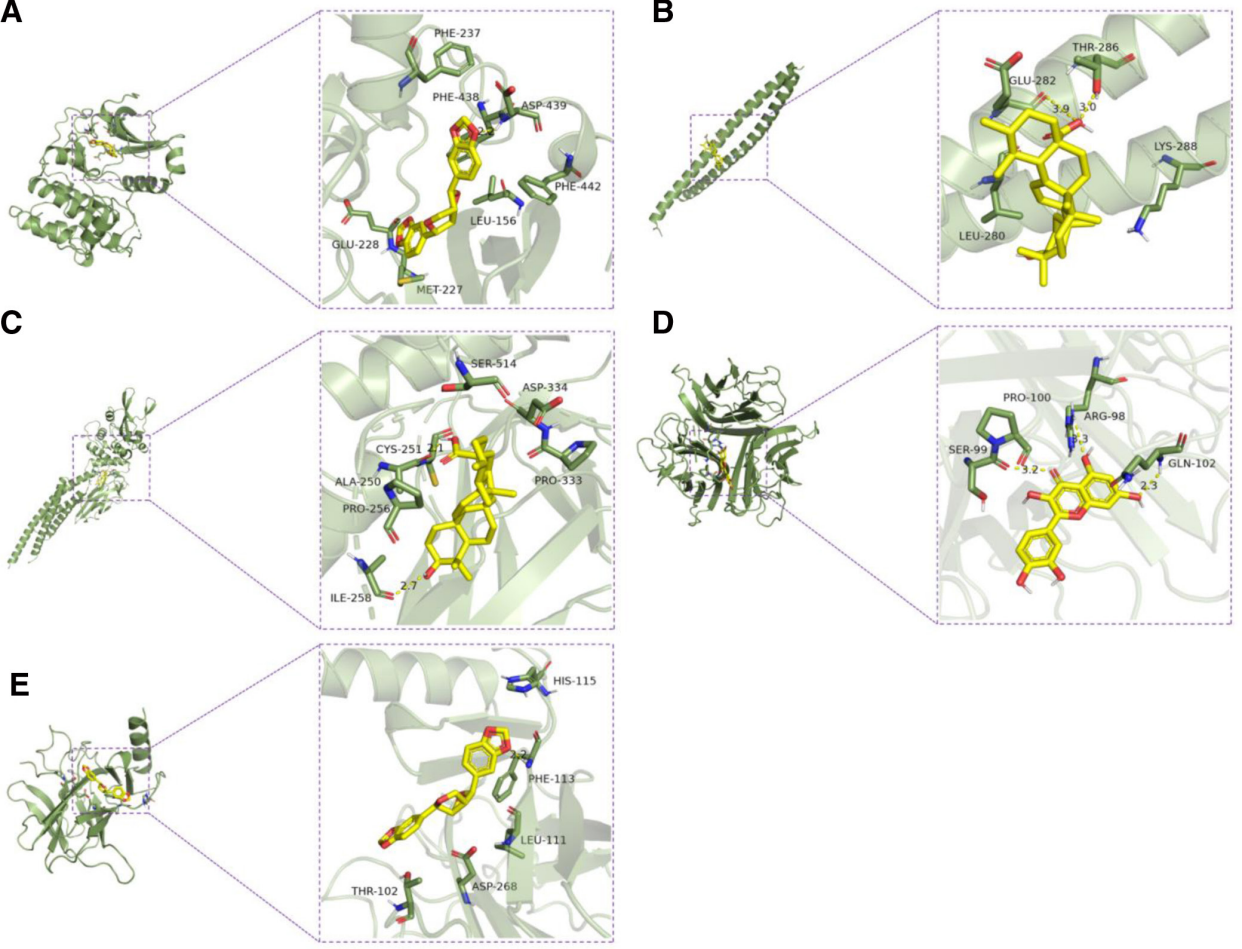
Molecular docking schemes of Acanthopanax senticosus targets and active components for vertigo treatment. (A) Interaction pattern analysis between sesamin and the *AKT1* protein. (B) Interaction pattern analysis between ursolic acid and the JUN protein. (C) Interaction pattern analysis between ursolic acid and the *STAT3* protein. (D) Interaction pattern analysis between quercetin and the *TNF* protein. (E) Interaction pattern analysis between sesamin and the *TP53* protein.

Docking results indicated that all small molecules could enter the active site of the target protein. The best-docking small molecule for each protein was selected and visualized in a diagram. As shown in Figure 7, sesamin formed a hydrogen bond with ASP-439 of *AKT1*, with a bond length of 2.2 Å. Ursolic acid forms hydrogen bonds with GLU-282 and THR-286 of JUN, with bond lengths of 3.9 Å and 3.0 Å, respectively. Ursolic acid forms hydrogen bonds with CYS-251 and ILE-258, with bond lengths of 2.1 Å and 2.7 Å, respectively. Quercetin forms hydrogen bonds with *TNF* GLN-102, ARG-98, and SER-99 with bond lengths of 2.3, 3.3, and 3.2 Å, respectively. Sesamin forms a hydrogen bond with *TP53* PHE-113 with a bond length of 2.2 Å. These small molecules also exhibit strong hydrophobic interactions with the surrounding amino acid residues. The detailed docking results are presented in the table. Sesamin achieved the highest docking score for *AKT1* and underwent kinetic simulation.

### 3.7. Results of MD

**Figure 8.**
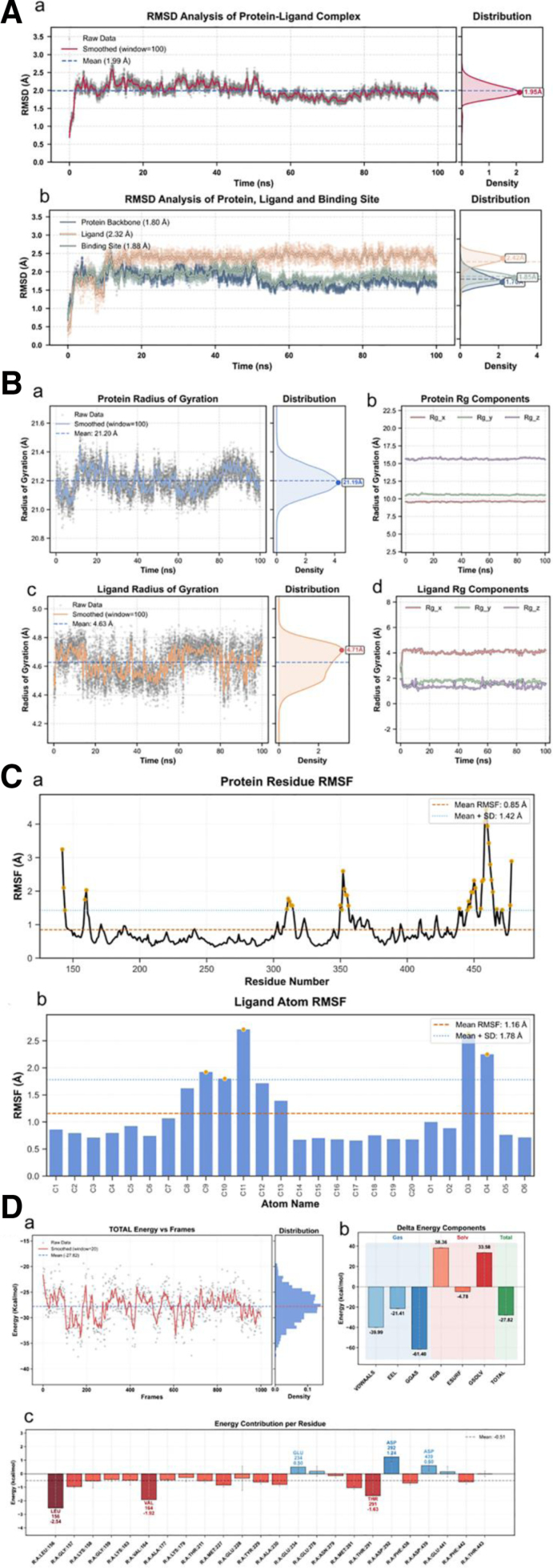
Simulation of Sesamin-*AKT1* dynamics. (A) RMSD results for Sesamin-*AKT1*. (A) Figure A displays the RMSD analysis results for the protein–ligand complex; b: RMSD plots for the protein backbone, ligand, and binding site are shown. The blue curve represents the RMSD of the protein backbone, the orange curve represents the RMSD of the ligand, and the green curve represents the RMSD of the binding site. (B) Sesamin and *AKT1* Rg results. (A) Changes in the protein radius of gyration (Rg) over simulation time, where gray scatter points represent raw data, the blue smoothed curve represents data with a window size of 100 ns, and the blue dashed line represents the average Rg value of 21.20 Å; (B) Evolution of the X, Y, and Z components of the protein Rg over time, represented by red, green, and blue curves, respectively; (C–D) Evolution of the ligand’s Rg and its components over time. (C) RMSF results for sesamin and *AKT1*. (A) RMSF analysis results for protein residues, with the X-axis labeled as protein residue numbers and the Y-axis representing residue RMSF values (unit: Å); b: RMSF analysis results for ligand atoms, with the X-axis labeled as atom names in the ligand and the Y-axis representing atom RMSF values (unit: Å). (D) RMSD Gibbs free energy between sesamin and *AKT1*. (A) The variation in total binding energy with the number of molecular dynamics simulation frames, including raw data (gray scatter plot), smoothed energy trajectory (red curve), and energy distribution histogram. b: Bar chart of delta energy components, displaying values for each energy component: van der Waals interactions (VDWAALS), electrostatic interactions (EEL), gas-phase free energy (GGAS), generalized Born polarizability (EGB), surface area-related energy (ESURF), solvation free energy (GSOLV), and total binding free energy (TOTAL). (C) Residue energy contribution plot, illustrating each residue contribution to the binding free energy. The gray dashed line indicates the average contribution value, with residues making the most significant energy contributions labeled. RMSD = root mean square displacement.

The docking results are presented in Table 2. Sesamin presented the highest docking score with *AKT1* and was selected for the kinetic simulations. As shown in Figure 8, root mean square displacement (RMSD) analysis revealed that the overall RMSD value of the protein–ligand complex in Figure 8A(a) exhibited significant fluctuations during the initial phase, but gradually stabilized as the simulation progressed, ultimately converging to approximately 2 Å. This finding indicated the relative structural stability of the complex throughout the simulation. Figure 8A(b) shows that the protein backbone RMSD averaged 1.80 Å, which was lower than both the ligand and binding site RMSDs, indicating greater backbone structural stability. The ligand exhibited the highest RMSD, averaging 2.32 Å, reflecting substantial conformational changes that potentially occur during binding. The RMSD of the binding site averaged 1.88 Å, indicating that despite ligand movement, the binding site remained relatively stable and played a crucial role in maintaining the overall stability of the complex.

**Table 2:** Docking results of core small molecules with core target proteins.

Targets	PDB ID	Active ingredient	Binding affinity (kcal/mol)	Targets	PDB ID	Active ingredient	Binding affinity (kcal/mol)
*TNF*	2E7A	Caffeic Acid	−6.8	*AKT1*	6NPZ	Caffeic Acid	−6.9
		Quercetin	−9.2			Quercetin	−8.5
		S-Adenosyl-L-Methionine	−8.7			S-Adenosyl-L-Methionine	−7.7
		Sesamin	−9.1			Sesamin	−9.5
		Ursolic Acid	−8.5			Ursolic Acid	−9.9
JUN	5T01	Caffeic Acid	−5.7	*STAT3*	6NJS	Caffeic Acid	−5.9
		Quercetin	−5.9			Quercetin	−7.2
		S-Adenosyl-L-Methionine	−5			S-Adenosyl-L-Methionine	−6.8
		Sesamin	−6.7			Sesamin	−7.9
		Ursolic Acid	−7.2			Ursolic Acid	−8.2
*TP53*	4AGP	Caffeic Acid	−5.8				
		Quercetin	−6.9				
		S-Adenosyl-L-Methionine	−6				
		Sesamin	−7.2				
		Ursolic Acid	−7.1				

In the Radius of Gyration (Rg) analysis, the Rg values of the protein and ligand in Figure 8B(a-d) exhibited minimal fluctuations throughout the simulation, indicating their relatively stable conformations. The average Rg of the protein was 21.20 Å, whereas that of the ligand was 4.63 Å. These values suggested that the protein adopts a moderately elongated ellipsoidal structure, whereas the ligand assumes a highly elongated linear conformation. As shown in Figure 8B(b) and Figure 8B(d), the Rg components along the protein axes show little variation, whereas the Rg_x component of the ligand is significantly larger than those of Rg_y and Rg_z, further confirming the highly elongated nature of the ligand. In the RMSF analysis, combining the results from Figure 8C(a) and Figure 8C(b), we observed that multiple regions within the protein, such as residues GLU459 and MET458, exhibited significant fluctuations. The high RMSF values in these regions may indicate flexible loops within the protein or loops for unbound ligands, which may play crucial roles in protein function. The fluctuation patterns of the ligand atoms revealed that certain atoms, such as C11 and O3, exhibit significant motion. This may indicate flexibility of the ligand during the binding process, potentially affecting binding stability. Correlation analysis between binding site residues and ligand atom fluctuation patterns suggested that fluctuations in binding site residues may influence the binding mode and stability of the ligand. These findings provide important insights into drug design and optimization, demonstrating that adjusting the flexibility or rigidity of ligands can increase their binding affinity and selectivity toward target proteins. In the RMSD Gibbs free energy analysis, Figure 8D(a) shows that the total binding energy remains relatively stable throughout the simulation, indicating a stable complex structure and energy equilibrium. In Figure 8D(b), van der Waals interactions and electrostatic interactions are the primary drivers of binding, with gas-phase interactions contributing more significantly to the binding free energy than solvation effects. Figure 8D(c) indicates that key residues such as R:A:LEU:156, R:A: VAL:164, and R:A:THR:291 significantly favored binding, whereas R:A:ASP:292 adversely affected binding. A total binding free energy (TOTAL) of -27.82 kcal/mol indicates a strong, favorable interaction between the protein and ligand. These results suggest that sesamin binds tightly to the stable structure of the *TP53* protein.

## 4. Discussion

Traditional Chinese medicine classifies vertigo under the category of “dizziness,” with treatment focused primarily on resolving blood stasis, unblocking meridians, and invigorating qi and blood circulation. Animal studies have demonstrated that Eleutherococcus senticosus prevents the reduction in weight and functional decline of the adrenal glands, thymus, spleen, liver, and kidneys observed during the “exhaustion” phase of the stress response.^[5]^ Its primary components, total flavonoids, dilate blood vessels, reduce blood viscosity, promote circulation, increase cerebral and cardiac blood flow, lower heart rate, and decrease tissue oxygen consumption and metabolic rate. It has anti-fatigue, anti-stress, and anti-inflammatory effects, exerts bidirectional regulatory effects on the central nervous system and leukocytes, enhances radiation resistance and immune function, scavenges oxygen free radicals, elevates human superoxide dismutase (SOD) levels, and promotes protein synthesis and tissue repair.^[5]^ In China, Acanthopanax senticosus has demonstrated clinical efficacy in treating vertigo, although its mechanism remains unclear. This study suggests that the primary active components of Acanthopanax senticosus are caffeic acid, quercetin, S-adenosyl-L-methionine, sesamin, and ursolic acid. Research indicates that caffeic acid exhibits anti-inflammatory, antioxidant, and antiferroptotic properties, improving ischemic brain injury in rats;^[6]^ quercetin alleviates neurotoxicity from cyclophosphamide-induced oxidative stress in rat brains by suppressing M1 polarization of microglia, thereby reducing neuroinflammation and oxidative stress to mitigate ischemic-reperfusion injury;^[7,8]^ S-adenosyl-L-methionine restores brain mitochondrial membrane fluidity, increases glutathione (GSH) levels, and inhibits brain lipid peroxidation, thereby mitigating oxidative stress damage;^[9,10]^ sesamin reduces oxidative stress and mortality in erythromycin-induced status epilepticus by inhibiting MEKs and cyclooxygenase (COX)-2 activation;^[11]^ and ursolic acid reduces ischemic-reperfusion injury in mouse brains postischemia by activating the Nrf2 pathway, mitigating oxidative stress and neuroinflammation.^[12,13]^These studies suggest that the active components in Eleutherococcus senticosus may directly and indirectly participate in regulating vertigo.

In these studies, *TP53*, *AKT1*, *STAT3*, *TNF*, JUN, SRC, HSP90AA1, IL-6, *EGFR*, and *NF-κB1* emerged as core therapeutic targets for the treatment of vertigo caused by Acanthopanax senticosus. *TP53* (tumor protein 53, p53) is one of the most important tumor suppressor genes in the human body. It encodes p53 protein, which plays a central role in cell cycle regulation, DNA repair, apoptosis, and tumor suppression. Research indicates that melatonin improves brain endothelial tight junction damage induced by hypoglycemic stress by inhibiting protein nitration of *TP53*-induced glycolytic and apoptotic regulators (TIGAR),^[14]^ 1,25-dihydroxyvitamin D3 alleviates ischemic brain injury by mitigating endoplasmic reticulum stress and ferroptosis by regulating vitamin D receptors and p53 signaling,^[15]^ and that, under oxygen-glucose deprivation/reperfusion (OGD/R) conditions, the overexpression of *TP53*-induced glycolytic and apoptotic regulator (TIGAR) increases the levels of phosphorylated mTOR and S6K p70, thereby mitigating ischemia/reperfusion-induced autophagy and ischemic brain injury.^[16]^ *AKT1* is a core molecule in the PI3K-AKT-mTOR signaling pathway and plays a crucial role in cell growth, proliferation, metabolism, and survival. Studies have shown that in in vivo models of cerebral ischemia, puerarin alleviates motor and cognitive deficits as well as hippocampal neuronal damage through the PI3K/*Akt1*/GSK-3β signaling pathway;^[17]^ nicotinamide mitigates cell death induced by focal cerebral ischemic injury, with neuroprotective effects mediated through the Akt signaling pathway, thereby promoting neuronal survival;^[18]^ and Sodium Danshen Su (SDSS) administration significantly reduces cerebral tissue necrosis and apoptosis induced by cerebral ischemia-reperfusion injury by activating the *AKT1* protein.^[19]^ Signal transduction and activation of transcription 3 (*STAT3*) is the core transcription factor of the JAK-STAT signaling pathway that regulates cell proliferation, differentiation, immune responses, and tumorigenesis. Sustained activation is associated with cancer, autoimmune diseases, and inflammatory conditions. Studies have demonstrated that inhibiting NLRP3 inflammasome activation by suppressing the JAK2/*STAT3* pathway improves ischemic stroke injury and neuroinflammation,^[20]^ inhibition of *STAT3*/NF-κB and inflammasome activation ameliorates neurological deficits and neuroinflammation in mice with traumatic brain injury,^[21]^ and modulating the *STAT3*/FOXO3a signaling pathway to suppress autophagy mitigates cerebral ischemia/reperfusion injury.^[22]^ *TNF*) is a key proinflammatory cytokine that is produced primarily by immune cells, such as macrophages and T cells, and plays a central role in inflammation, immune regulation, apoptosis, and tumorigenesis. Research has demonstrated that *TNF*-α pretreatment enhances the survival and function of transplanted human neural progenitor cells following hypoxic-ischemic brain injury.^[23]^ By regulating microglial polarization and neuroinflammation mediated by the cGAS/STING/NF-κB pathway, *TNF*-α pretreatment mitigates brain injury caused by subarachnoid hemorrhage.^[24]^ Inhibiting of TLR4/NF-κB signaling reduces neuroinflammation and glial autophagy, thereby improving brain injury-induced by heatstroke.^[25]^ JUN (c-Jun) is a core member of the AP-1 (activator protein-1) transcription factor family and belongs to the proto-oncogene category. It plays a pivotal role in cellular proliferation, differentiation, apoptosis, and stress response. Abnormal activation is associated with various cancers, inflammatory diseases, and fibrosis. Research has demonstrated that, in both in vivo and in vitro models, the overexpression of S1pr2 in endothelial cells following traumatic brain injury promotes blood-brain barrier disruption via the c-Jun N-terminal kinase (JNK)/c-Jun/MMP-9 pathway,^[26]^ and the inhibition of JNK mitigates early brain injury-induced neuronal apoptosis in subarachnoid hemorrhage rats by reducing p53 phosphorylation and the activation of the mitochondrial apoptosis pathway.^[27]^ Additionally, SRC,^[28]^ HSP90AA1,^[29]^ IL-6,^[30]^ *EGFR*,^[31]^ and *NF-κB1*^[31]^ participate in regulating brain injury. These studies suggest that *TP53*, *AKT1*, *STAT3*, *TNF*, JUN, SRC, HSP90AA1, IL-6, *EGFR*, and *NF-κB1* may be directly and indirectly involved in the regulatory mechanisms of Eleutherococcus senticosus treatment of vertigo.

KEGG pathway analysis revealed that the IL-17, *TNF*, PI3K–Akt, p53, HIF-1, and FoxO signaling pathways are key pathways involved in the treatment of vertigo with Acanthopanax senticosus. The IL-17 signaling pathway serves as a pivotal link between inflammation and immune responses. The IL-17 family of cytokines (e.g., IL-17A-F), which are secreted by Th17 cells and their receptors (IL-17RA-RE), play critical roles in autoimmune diseases, infection defense, and tumor microenvironment regulation. Studies have revealed that CD4^+^T cells disrupt the integrity of the blood-brain barrier by releasing interleukin-17, thereby promoting the progression of perihematomal edema;^[32]^ IL-17A may exacerbate cerebral ischemia/reperfusion (I/R) injury by increasing calpain-mediated proteolysis of the TRPC6 protein;^[33]^ in adult mice, it promotes surgery-induced neuroinflammation, microglial phagocytosis, and cognitive impairment via the IL-17/CCAAT enhancer binding proteins beta/C3 pathway;^[34]^ IL-17 exacerbates oxygen–glucose deprivation-induced neuronal injury and modulates IL-17 receptor expression in neurons.^[35]^ The *TNF* signaling pathway is a central pathway that regulates inflammation, immune modulation, and cell survival. It is mediated by tumor necrosis factor (*TNF*-α) and its receptors (*TNF*R1/*TNF*R2) and is closely associated with autoimmune diseases, infections, tumors, and metabolic disorders. Studies have shown that inhibition of the *TNF*-α/NF-κB pathway improves doxorubicin-induced cortical and hippocampal brain damage.^[36]^ Following experimental traumatic brain injury, tumor necrosis factor-α impairs perivascular cell-mediated cerebral microcirculation via the nuclear factor-κB/inducible nitric oxide synthase axis.^[37]^ The miR-27a/Muscle RING Finger 1/*TNF*-α and mitochondrial apoptosis pathways participate in apoptosis induced by cerebral ischemia–reperfusion injury in rats.^[38]^ The PI3K–Akt signaling pathway serves as a central signaling network that regulates key physiological processes, such as metabolism, proliferation, survival, growth, and angiogenesis within cells. Research has indicated that modulating the PI3K/Akt/NF-κB signaling pathway promotes M2 polarization of microglia/macrophages, thereby mitigating cerebral ischemia/reperfusion injury.^[8]^ Following cerebral hemorrhage in mice, TREM2 activation mitigates neuroinflammation and neuronal apoptosis by modulating the PI3K/Akt pathway;^[39]^ modulation of the PI3K/AKT/Foxo1 signaling pathway reduces GSDMD-mediated microglial pyroptosis in spinal cord injury;^[40]^ and inhibition of autophagy via activation of the PI3K/AKT/mTOR pathway alleviates cerebral ischemia–reperfusion injury.^[41]^ The p53 signaling pathway is a critical tumor suppression network within cells. Sensing cellular stress signals, such as DNA damage and oxidative stress, regulates cell cycle arrest, DNA repair, and apoptosis to maintain genomic stability. Studies have shown that reducing p53 phosphorylation levels and activating the mitochondrial apoptosis pathway in rats with subarachnoid hemorrhage alleviates EBI-induced neuronal apoptosis.^[27]^ In ICR mouse brains and PC12 cells, excessive Mn exposure induces ferroptosis via the HIF-1α/p53/Solute carrier family 7 member 11 pathway.^[42]^ The *TP53*/PINK1/PRKN pathway accelerates mitochondrial autophagy during cerebral I/R injury.^[43]^ HIF-1 (hypoxia-inducible factor-1) is a transcription factor that is activated under hypoxic conditions and is composed of HIF-1α (inducible subunit) and HIF-1β (constitutive subunit), which regulate adaptive cellular responses to hypoxic environments. Research indicates that cumin seed extract mitigates cerebral ischemia/reperfusion injury by activating the HIF-1α/vascular endothelial growth factor A pathway,^[44]^ which promotes cerebral microvascular angiogenesis through the HIF-1α-vascular endothelial growth factor A-Notch1 signaling pathway, thereby protecting against cerebral ischemia-reperfusion injury;^[45]^ and HIF-1α gene knockdown reduces inflammation and oxidative stress in male rats with ischemic stroke via the CXCR4/NF-κB pathway.^[45]^ FoxO is a family of transcription factors that plays crucial roles in cell cycle regulation, apoptosis, metabolic homeostasis, and oxidative stress responses. Studies have shown that inhibiting FoxO3a can alleviate neurobehavioral deficits following traumatic brain injury by suppressing neuronal autophagy,^[46]^ granulocyte chemotactic protein-2 regulates cellular permeability, proliferation, and apoptosis following ischemia–reperfusion injury by modulating Sirt3 expression through activation of the AKT/FOXO3a pathway,^[47]^ and in adult rats, delayed hypoxia posttreatment contributes to inducing neuroprotection against transient global cerebral ischemia by activating the Akt/FoxO pathway and inhibiting the MEK/ERK pathway.^[48]^ These studies suggest that the IL-17, *TNF*, PI3K–Akt, p53, HIF-1, and FoxO signaling pathways may directly and indirectly participate in the regulatory mechanisms of Eleutherococcus senticosus treatment for vertigo.

Molecular docking revealed that sesamin has a strong affinity for *AKT1* and *TP53*, quercetin has a strong affinity for *TNF*, and ursolic acid has a strong affinity for *STAT3* targets. These findings suggest that sesamin, quercetin, and ursolic acid may be key active components in the regulatory pathways of Acanthopanax senticosus in the treatment of vertigo. JUN, *TNF*, *AKT1*, *STAT3*, and *TP53* are the core regulatory targets in this therapeutic mechanism. Sesamin had the highest docking score for the *AKT1*. MDs revealed high stability of the protein–ligand complex throughout the simulation via RMSD, RMSF, Rg, and hydrogen bond analyses. The negative free energy (−37.78 kcal/mol) further confirmed strong protein–ligand binding, confirming the structural stability and binding mode of the complex.

## 5. Conclusion

This study elucidates JUN, *TNF*, *AKT1*, *STAT3*, and *TP53* as core regulatory targets in Acanthopanax senticosus treatment for vertigo, while sesamin, quercetin, and ursolic acid may represent key active components in this therapeutic approach. The IL-17, *TNF*, PI3K-Akt, p53, HIF-1, and FoxO signaling pathways may all participate in the regulation of Acanthopanax senticosus treatment for vertigo, with the *TNF* and PI3K-Akt signaling pathways likely serving as core regulatory pathways. This study provides a reference for further in vivo and in vitro studies.

## Acknowledgments

This work was supported by the Yunnan Applied Basic Research Project-Union Foundation of China under Grant No. 202201AY070001--091.

## Author contributions

**Conceptualization:** Rui-Qiong Ba, Ping Li, Hen-Jie Zhu, Shu-Ji Gao, Yan-Lin Zhu, Ming-Wei Liu.

**Investigation:** Rui-Qiong Ba, Jin-Mei Ning, Xiang Ma, Shu-Ji Gao, Yan-Lin Zhu, Ming-Wei Liu.

**Software:** Rui-Qiong Ba, Jin-Mei Ning, Xiang Ma, Hen-Jie Zhu, Shu-Ji Gao.

**Data curation:** Jin-Mei Ning, Yao Cui, Hen-Jie Zhu, Shu-Ji Gao.

**Project administration:** Jin-Mei Ning, Yan-Lin Zhu, Ming-Wei Liu.

**Validation:** Jin-Mei Ning, Xiang Ma, Hen-Jie Zhu, Ming-Wei Liu.

**Formal analysis:** Ping Li, Xiang Ma, Yan-Lin Zhu.

**Methodology:** Ping Li, Yao Cui, Hen-Jie Zhu.

**Funding acquisition:** Yao Cui, Ming-Wei Liu.

**Supervision:** Yao Cui, Yan-Lin Zhu, Ming-Wei Liu.

**Resources:** Hen-Jie Zhu.

**Visualization:** Yan-Lin Zhu.

**Writing – original draft:** Rui-Qiong Ba, Ping Li, Xiang Ma, Yao Cui, Shu-Ji Gao, Ming-Wei Liu.

**Writing – review & editing:** Ming-Wei Liu.
